# Accurate Evaluation of Electro-Thermal Performance in Silicon Nanosheet Field-Effect Transistors with Schemes for Controlling Parasitic Bottom Transistors

**DOI:** 10.3390/nano14121006

**Published:** 2024-06-10

**Authors:** Jinsu Jeong, Sanguk Lee, Rock-Hyun Baek

**Affiliations:** Department of Electrical Engineering, Pohang University of Science and Technology (POSTECH), Pohang 37673, Republic of Korea; js.jeong@postech.ac.kr (J.J.); sanguk96@postech.ac.kr (S.L.)

**Keywords:** electro-thermal performance, gate-all-around, lattice temperature, nanosheet FET, parasitic bottom transistor, ring oscillator, self-heating effect, sub-3 nm node, TCAD simulation, trench inner-spacer

## Abstract

The electro-thermal performance of silicon nanosheet field-effect transistors (NSFETs) with various parasitic bottom transistor (*tr_pbt_*)-controlling schemes is evaluated. Conventional punch-through stopper, trench inner-spacer (TIS), and bottom oxide (BOX) schemes were investigated from single-device to circuit-level evaluations to avoid overestimating heat’s impact on performance. For single-device evaluations, the TIS scheme maintains the device temperature 59.6 and 50.4 K lower than the BOX scheme for n/pFETs, respectively, due to the low thermal conductivity of BOX. However, when the over-etched S/D recess depth (*T_SD_*) exceeds 2 nm in the TIS scheme, the RC delay becomes larger than that of the BOX scheme due to increased gate capacitance (*C_gg_*) as the *T_SD_* increases. A higher TIS height prevents the *C_gg_* increase and exhibits the best electro-thermal performance at single-device operation. Circuit-level evaluations are conducted with ring oscillators using 3D mixed-mode simulation. Although TIS and BOX schemes have similar oscillation frequencies, the TIS scheme has a slightly lower device temperature. This thermal superiority of the TIS scheme becomes more pronounced as the load capacitance (*C_L_*) increases. As *C_L_* increases from 1 to 10 fF, the temperature difference between TIS and BOX schemes widens from 1.5 to 4.8 K. Therefore, the TIS scheme is most suitable for controlling *tr_pbt_* and improving electro-thermal performance in sub-3 nm node NSFETs.

## 1. Introduction

Silicon fin field-effect transistors (FinFETs) have been successfully scaled down to 3 nm nodes and have gained acceptance in the logic foundry industry [1]. The downscaling made the fin channels thinner and taller to maintain electrostatics and performance. Nevertheless, the significantly heightened fin aspect ratio resulted in a structural instability of the fins, leading to their bending and breaking [2,3]. Furthermore, the number of fins per device was depopulated from 3 to 1 fin to reduce the cell height. Performance modulation of FinFETs mainly depends on adjusting the number of fins; therefore, fin depopulation is a challenge for circuit design with FinFETs. To overcome the limitations of FinFETs, vertically stacked silicon gate-all-around nanosheet (NS) FETs have emerged as a replacement for FinFETs [4,5]. In contrast to FinFETs, NS channels are laterally wide, resulting in a low aspect ratio and stable structure [5]. In addition, continuous performance modulation is possible by adjusting the NS width, which enables flexible circuit design.

Regrettably, the distinctive structure of NSFETs leads to a parasitic bottom transistor (*tr_pbt_*) that forms beneath the bottommost NS channel [6,7,8]. The *tr_pbt_* is a critical failure factor that causes significant punch-through leakage because gate controllability is not achieved in the *tr_pbt_*. Conventionally, a counter-doped punch-through stopper (PTS) layer has been used to control the leakage in *tr_pbt_* [9]. However, the PTS loses its ability to control the leakage if the source/drain (S/D) recess is unintentionally over-etched [6,10].

**Figure 1:**
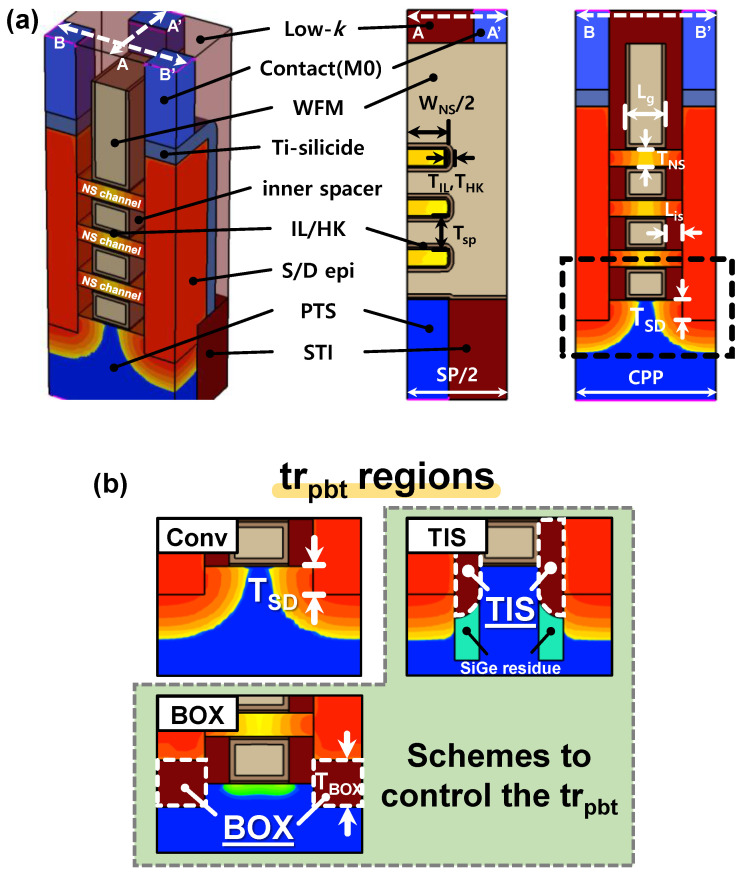
(**a**) Three-dimensional structure and cross-sections of Conv-NSFETs, and (**b**) structural comparison of the Conv, TIS, and BOX schemes.

The insertion of dielectric layers between the S/D and the substrate, known as the bottom oxide (BOX) scheme, is one of the commonly known methods to control the *tr_pbt_* [10,11]. BOX acts as an etch-stop layer that regulates the S/D recess depth variations. In addition, BOX allows for the thorough blocking of the leakage path. Alternatively, the trench inner-spacer (TIS) scheme was proposed in our previous study to control *tr_pbt_*, even under over-etched S/D recess conditions [12]. In the TIS scheme, the bottom inner-spacer is extended toward the PTS layer, preventing S/D dopants from diffusing into the channel of the *tr_pbt_* (see Figure 1 for details). The TIS scheme is immune to variation in the over-etched S/D recess depth (*T_SD_*) because the TIS height is always higher than the *T_SD_*.

The main difference between the BOX and TIS schemes is the presence of an oxide layer between the S/D and the substrate. As the heat generated in the channels is primarily dissipated from the drain toward the substrate, BOX with a lower thermal conductivity (*κ*) than silicon hinders the heat dissipation [12,13]. This implies that the temperature of the device differs depending on the scheme used. Consequently, the electro-thermal performance may also vary, taking into account the self-heating effect (SHE).

Previous studies have compared the electro-thermal characteristics considering the schemes used to control the *tr_pbt_*, but they have some limitations. First, some studies compared the electro-thermal performance between conventional (Conv) and BOX schemes [14,15]. However, one of them focused solely on the evaluation of the heat dissipation capability without considering the effects of heat on electrical performance. The other study primarily examined the operation of a single device in steady-state DC and neglected the heating and cooling cycles of the devices. As a result, the effects of heat on electrical performance may be overestimated. In contrast, a comparison between TIS and BOX schemes by considering the heating and cooling cycles was investigated in our previous work [12]. However, only the ability to dissipate heat during single-device operations was evaluated without quantitatively investigating the effects of heat on electrical performance. In addition, no circuit-level evaluation was conducted to reflect the actual behavior of the system-on-chip (SoC) product.

Therefore, an accurate and comprehensive evaluation of the electro-thermal performance in sub-3 nm node NSFETs with the Conv, TIS, and BOX schemes for the single-device-to-circuit level is conducted in this study. First, the device structures and simulation methods are presented in Section 2. Secondly, Section 3.1 examines the electro-thermal performance at the single-device level in steady state. Finally, in Section 3.2, the electro-thermal performance of a ring oscillator (RO) is compared based on the transient characteristics using a 3D mixed-mode simulation. All characteristics are based on fully calibrated Synopsys Sentaurus Technology Computer-Aided Design (TCAD) simulations [16,17].

## 2. Simulation Structure and Methodology

The TCAD simulation framework for 3-stacked silicon NSFETs was defined as follows. Both process and device simulations were performed using the Sentaurus process and device simulator [16,17]. First, we benchmarked the NSFET structures in [4] and calibrated the simulated data and parameters to match the measured transfer curves, as described in our previous work [12]. Subsequently, the NSFETs were scaled down to specifications suitable for sub-3 nm node, and then schemes were applied to control the *tr_pbt_*. The PTS layer was doped with 2 × 10^18^ cm^−3^ in the Conv and TIS schemes, but undoped in the BOX scheme [11].

Figure 1 presents detailed NSFET structures with the Conv, TIS, and BOX schemes, where the regions with *tr_pbt_* are highlighted. Geometric and device parameters are listed in Table 1. The TIS and BOX schemes each include an additional process step compared to the Conv scheme: trench patterning prior to the Si and sacrificial layer (SiGe_0.3_) epi stacking for the TIS scheme [12], and oxide deposition before the S/D epi growth for the BOX scheme. Here, The TIS scheme leaves SiGe_0.3_ residues under the TIS, and the BOX thickness (*T_BOX_*) was determined as 10 nm. Various *T_SD_* values were considered in the Conv and TIS schemes to thoroughly verify the effects of *tr_pbt_*.

For the device and circuit simulations, a comprehensive set of physical models was integrated to simulate various aspects of semiconductor behavior. A self-consistent hydrodynamic model was used to ensure accurate carrier and energy transport. This also facilitated the calculation of device temperatures, which reflect the thermal effects on device performance [18]. The inversion and accumulation layer model was included to account for Coulomb, phonon, and scattering from rough surfaces for carrier mobility. In addition, low-field ballistic mobility was included to account for an additional contribution to low-field mobility for devices with very short channels. The low-field mobility model was adjusted so that the simulated data matched the measured data. The Shockley–Read–Hall and Auger models considered recombination and generation, while the Hurkx model was implemented for band-to-band tunneling. To account for quantum mechanical effects, the density gradient model was used. Furthermore, the deformation potential model was included to consider strain-induced changes in the band structure, and the Slotboom model was incorporated to analyze the effects of doping on bandgap narrowing.

*κ* of semiconductor regions was calculated based on the Boltzmann transport equations for phonons with relaxation time approximation. In this context, dependencies of *κ* on physical size, doping types and concentration, alloy composition, and lattice temperature were considered [19,20,21,22,23]. The Sentaurus device simulator provides built-in functions for a self-consistent calculation of *κ*, and the *κ* values at 300 K are summarized in Table 2. For the thermal boundary condition, the adiabatic sidewalls along with the contact thermal resistivities of the substrate (*r_th_*_,*sub*_) and the BEOL (*r_th_*_,*BEOL*_) to be connected to the heat sink at 300 K were assumed. The *r_th_*_,*sub*_ and *r_th_*_,*BEOL*_ were calculated on a Si substrate with dimensions of 5 × 5 μm^2^ (area), a thickness of 50 μm, and a BEOL height of 1 μm (contact-M10).

## 3. Results and Discussion

### 3.1. Evaluation of Electro-Thermal Performance of Single-Device Operations under Steady State

**Figure 2:**
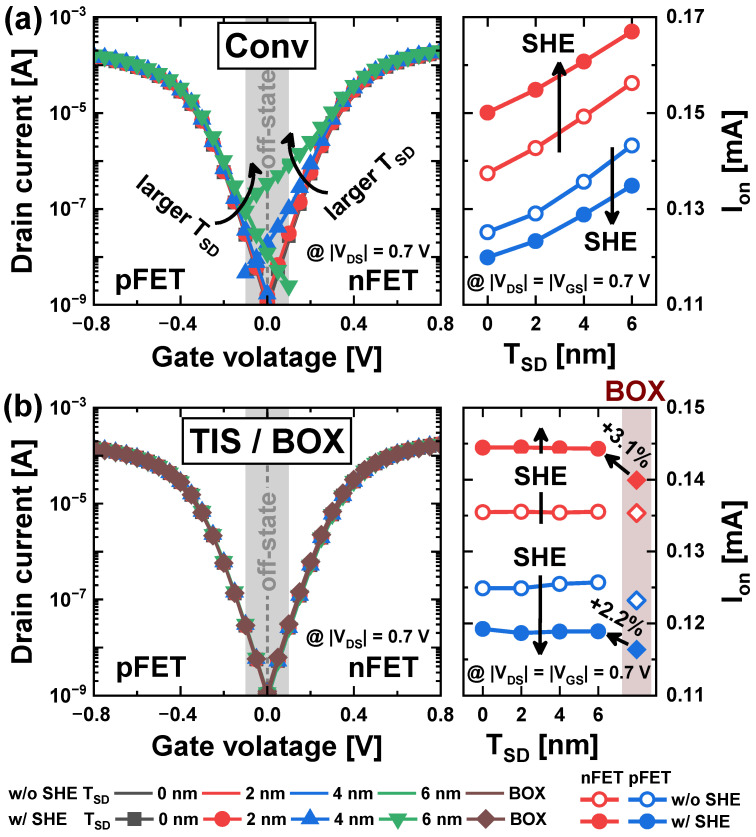
Transfer characteristics and *I_on_* for various *T_SD_* with and without SHE in (**a**) Conv, (**b**) TIS, and BOX schemes (the transfer curves with and without SHE almost overlaps).

The transfer curves of Conv-NSFETs as a function of different *T_SD_* are presented in Figure 2a. Here, the off-state current (*I_off_*) at *T_SD_* = 0 nm was fixed to 1 nA to allow for a fair comparison. The *I_off_* was defined as the drain current (*I_ds_*) at *V_GS_* = 0 V and |*V_DS_*| = 0.7 V. The Conv scheme shows a pronounced increase in *I_off_* as the *T_SD_* increases. In particular, for nFETs with *T_SD_* ≥ 4 nm, and for pFETs with *T_SD_* ≥ 6 nm, *I_off_* becomes 10 times larger than that of *T_SD_* = 0, resulting in critical failures in the SoC products [6,24]. On-state current (*I_on_*) also increases with increasing *T_SD_* as the leakage current in the *tr_pbt_* contributes to *I_on_* (*I_on_* was defined as *I_ds_* at |*V_GS_*| = |*V_DS_*| = 0.7 V). In contrast, the TIS scheme completely controls the *tr_pbt_*, even in deep *T_SD_*, by preventing S/D dopants from diffusing into the channel of the *tr_pbt_* (Figure 2b) [12]. Thus, the *I_on_* in the TIS scheme is not affected by the *T_SD_* variations. The *T_SD_* variations do not occur in the BOX scheme, and the *tr_pbt_* has no effect on the *I_on_*.

Furthermore, Figure 2 shows the effects of the SHE on the *I_ds_*. In all three schemes, the *I_off_* is rarely affected by SHE, but the *I_on_* is affected. This is attributed to the negligible heat generation in the off-state, which is due to the low current level. In contrast, *I_on_* is large enough to cause joule-heating. There are two points of analysis regarding the SHE in Figure 2: (1) the opposite *I_on_* change trend caused by the SHE in the n/pFET, and (2) the comparison of *I_on_* in the Con, TIS, and BOX schemes under the SHE.

**Figure 3:**
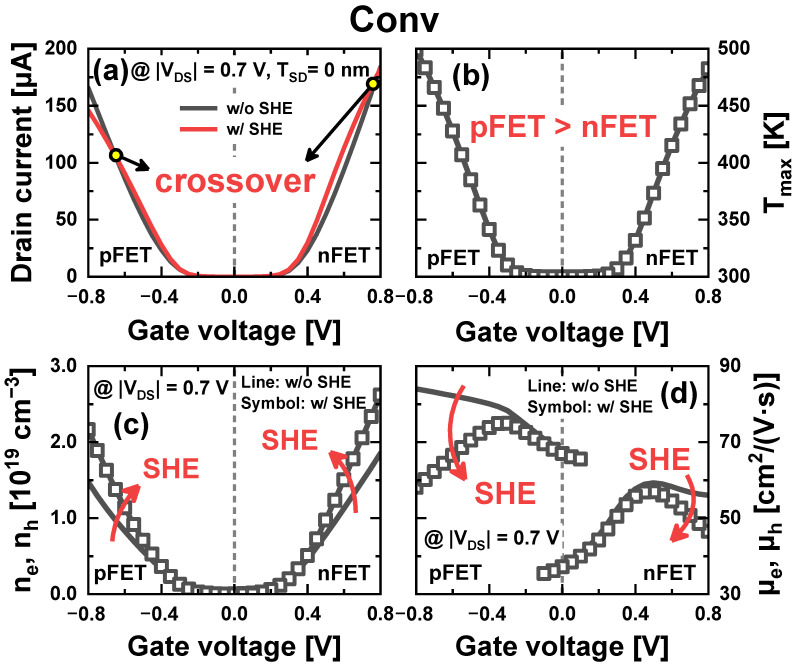
(**a**) Transfer characteristics of NSFETs with and without SHE, and (**b**) *T_max_* in the Conv scheme. (**c**) Carrier density and (**d**) mobility with or without SHE in the Conv scheme.

First, in all schemes, the *I_on_* under the SHE (*I_on_SHE_*) is larger than that without SHE (*I_on_noSHE_*) in nFETs, whereas it is smaller in pFETs (Figure 3a). This is because the maximum lattice temperature (*T_max_*) in pFETs is higher than that in nFETs due to the smaller *κ* of SiGe_0.5_ S/D than that of SiC_0.02_ S/D (Figure 3b). An increase in the lattice temperature leads to two opposing phenomena in *I_on_*: (1) an increase in carrier density (*n_e_*, *n_h_*) and (2) a reduction in carrier mobility (*μ_e_*, *μ_h_*) (Figure 3c,d). The increase in carrier density is because the elevated *T_max_* results in an increased intrinsic carrier density. The reduction in carrier mobility is caused by increased phonon scattering. The increase in carrier density is dominant at a low *V_GS_* and the reduction in carrier mobility is dominant at a high *V_GS_*. A crossover point (*V_co_*) then occurs, at which the magnitudes of *I_ds_SHE_* and *I_ds_noSHE_* are reversed. *V_co_* is defined as the *V_GS_* at which *I_ds_SHE_* = *I_ds_noSHE_*.

In Figure 3a, *I_ds_SHE_* is larger than *I_ds_noSHE_* when the *V_GS_* is smaller than the *V_co_*. This is because the increased carrier density due to the elevated *T_max_* dominates over the reduction in carrier mobility for *V_GS_* ≤ *V_co_*. For the *V_GS_* ≥ *V_co_*, however, the *I_ds_SHE_* becomes smaller as the contributions of these two factors are reversed. Here, both *μ_e_* and *μ_h._* have almost the same temperature dependence [25], and the *T_max_* of pFET is larger than that of nFETs. Thus, the reduction in *μ_h_* in pFET is significantly larger than *μ_e_* in nFET. As a result, *V_co_* was determined at a lower *V_GS_* in pFETs than in nFETs (0.8 V for nFETs and −0.65 V for pFETs). Therefore, the *I_on_SHE_* is larger than the *I_on_noSHE_* nFETs, whereas it is smaller in pFETs.

Secondly, the three schemes show different trends of *I_on_* with respect to the *T_SD_* (Figure 2). The *I_on_* in the Conv scheme increases linearly with deeper *T_SD_* as the leakage current through the *tr_pbt_* contributes to *I_on_*. However, the *I_on_* in the TIS scheme hardly varies with the *T_SD_* due to the suppression of the leakage in *tr_pbt_*. Moreover, for nFETs, the TIS scheme has a 3.1% higher *I_on_SHE_* value compared to the BOX scheme, although both schemes have almost the same *I_on_noSHE_* value. Similarly, for pFETs, the TIS scheme has a 2.2% higher *I_on_SHE_* value than the BOX scheme.

**Figure 4:**
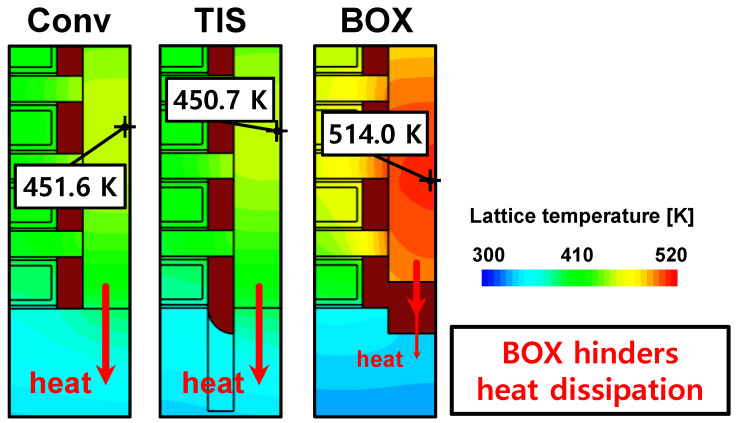
Lattice temperature profiles at on-state in the Conv, TIS, and BOX schemes. The marked points indicate *T_max_*.

**Figure 5:**
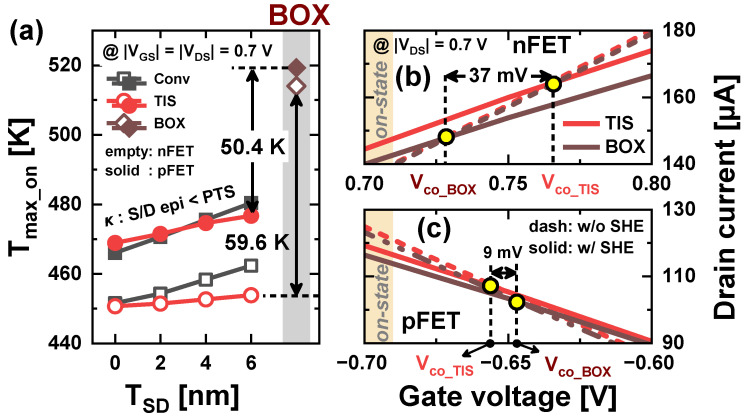
(**a**) *T_max_on_* compared to different *T_SD_* in Conv, TIS, and BOX schemes. (**b**,**c**) *V_co_* comparison in TIS and BOX schemes for n/pFETs.

The reason for the TIS and BOX schemes showing different changes in *I_on_SHE_* is attributed to the different *T_max_* values. Conv and TIS schemes with *T_SD_* = 0 nm have similar *T_max_* because the heat can dissipate unhindered through the substrate (Figure 4). The similarity in *T_max_* between the TIS and Conv schemes is an advantage of the TIS scheme, as it means that the TIS scheme can control the impact of *tr_pbt_* without affecting thermal performance. However, the lower *κ* of BOX compared to the S/D epitaxy hinders heat dissipation, causing the BOX scheme to have a significantly higher *T_max_* than the others. Furthermore, *T_max_* in the on-state (*T_max_on_*) increases linearly with the larger *T_SD_*, as the S/D epitaxies with the lower *κ* than PTS intrudes the PTS (Figure 5a). Nevertheless, the BOX scheme in n/pFETs still exhibits 59.6 and 50.4 K higher *T_max_on_* compared with those of the TIS scheme with *T_SD_* = 6 nm, respectively. As a result, the reduction in carrier mobility due to the SHE is greater in the BOX scheme than in the TIS scheme. Especially in nFETs, the *V_co_* in the BOX scheme is significantly lower than that in the TIS scheme due to the *T_max_* difference (Figure 5b,c). Therefore, the increase in *I_on_* due to the SHE is more pronounced in the TIS scheme than in the BOX scheme, resulting in a higher *I_on_SHE_* of the TIS scheme for nFETs.

**Figure 6:**
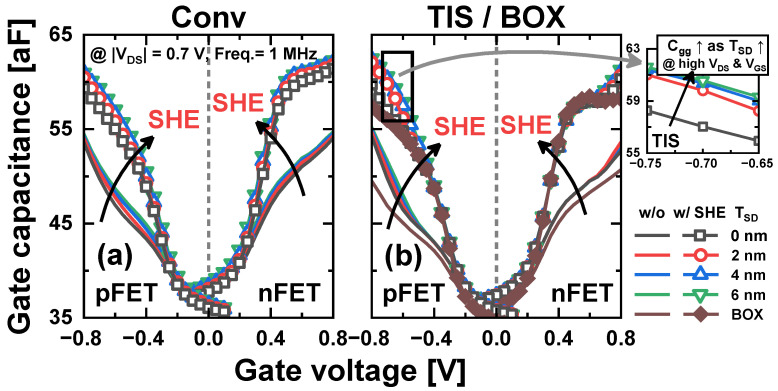
*C_gg_*-*V_GS_* curves for different *T_SD_* with and without SHE in (**a**) Conv, (**b**) TIS, and BOX schemes (inset: *C_gg_* at high and *V_GS_* in the TIS scheme).

**Figure 7:**
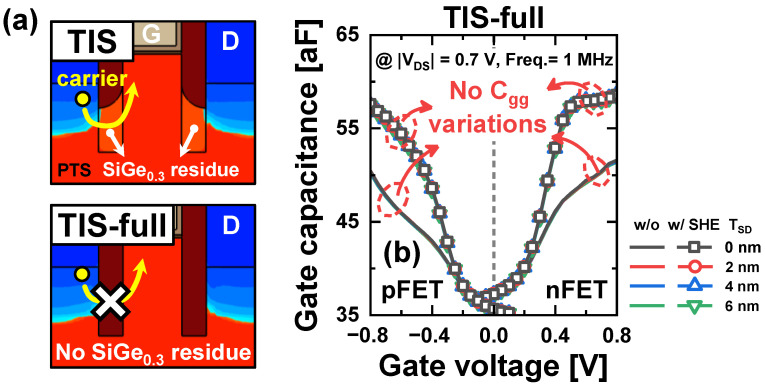
(**a**) Structural comparison of the TIS-full scheme and the TIS scheme; (**b**) *C_gg_*-*V_GS_* curves for different *T_SD_* with and without SHE in the TIS-full scheme.

**Figure 8:**
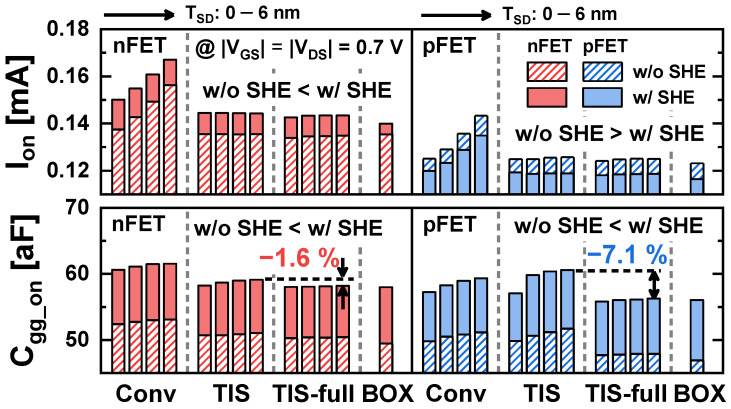
Comparison of *I_on_* and *C_gg_on_* in the Conv, TIS, TIS-full, and BOX schemes.

Figure 6 shows the gate capacitance (*C_gg_*) as a function of *V_GS_* in Conv, TIS, and BOX schemes. As increased intrinsic carriers respond to the small signal of the gate due to elevated *T_max_*, the SHE increases the *C_gg_* in all schemes. In the Conv scheme, the *C_gg_* increase with a large *T_SD_* is also due to the increased inversion capacitance of the *tr_pbt_* (Figure 6a) [6]. In contrast, in the TIS scheme, *C_gg_* is not dependent on the *T_SD_* at low *V_GS_*. However, at high *V_GS_*, *C_gg_* increases with a large *T_SD_* because carriers in the source are pulled toward the PTS and respond to the small signal of the gate (inset of Figure 6b) [12]. For this reason, the formation of a deeper TIS allows for a reduction in *C_gg_*. The TIS scheme inevitably leaves residues of the SiGe_0.3_ sacrificial layer beneath the TIS during the isotropic selective etch of the sacrificial layer for inner-space formation because the depth of the trench is larger than the height of the TIS [12]. By performing an additional cleaning step to remove the SiGe_0.3_ residues within the trench, the TIS can be formed to a depth equal to that of the trench. This configuration is referred to as the TIS-full scheme (Figure 7a). In the TIS-full scheme, the *C_gg_* does not rely on the *T_SD_*, as shown in Figure 7b. Therefore, the TIS-full scheme is also used only for a comparison. The *I_on_* and *C_gg_* in the on-state (*C_gg_on_*) in the four schemes are summarized in Figure 8. The TIS-full scheme has almost the same *I_on_* as the TIS scheme. However, *C_gg_on_* is reduced by up to 1.6% and 7.1% in the TIS-full scheme compared with the TIS scheme for n/pFETs.

**Figure 9:**
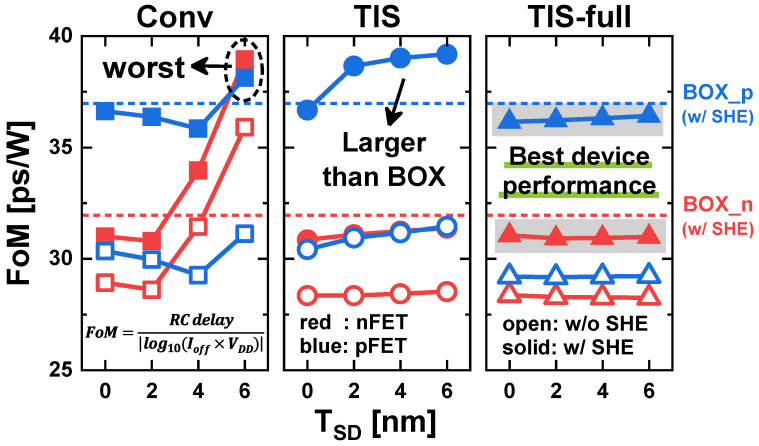
Figure-of-merit (FoM) comparison for Conv, TIS, TIS-full, and BOX schemes.

For a fair performance comparison, it is necessary to account for static power consumption. Figure-of-merit (FoM) is defined as RC delay/|log_10_(*I_off_* × *V_DD_*)|, where RC delay = *C_gg_on_* × *V_DD_*/*I_on_*. The reason that log_10_ is taken for the static power term is that the contribution of static power should not be overestimated. The smaller the FoM, the better the electrical performance. Figure 9 illustrates the FoM as a function of the *T_SD_* and SHE in the four schemes. First, in the Conv scheme, the FoM increases significantly with a large *T_SD_* due to the leakage current, which spikes sharply with increasing *T_SD_*. As a result, the Conv scheme exhibits the worst device performance with *T_SD_* = 6 nm. Second, the FoM in the TIS scheme does not vary much with larger *T_SD_*, except for the pFETs under the SHE. The pFETs with *T_SD_* = 0 nm show a smaller FoM than the BOX scheme, but it increases significantly as the *T_SD_* becomes larger due to increased *C_gg_* (Figure 6b). Third, the FoM in the TIS-full scheme is not varied by the *T_SD_*, and is consistently smaller than that of the BOX scheme regardless of the *T_SD_*. Consequently, the TIS-full scheme with the smallest FoM shows the best electro-thermal performance in single-device operation under steady-state conditions.

### 3.2. Electro-Thermal Performance of the Three-Stage Ring Oscillator in the 3D Mixed-Mode Simulation

**Figure 10:**
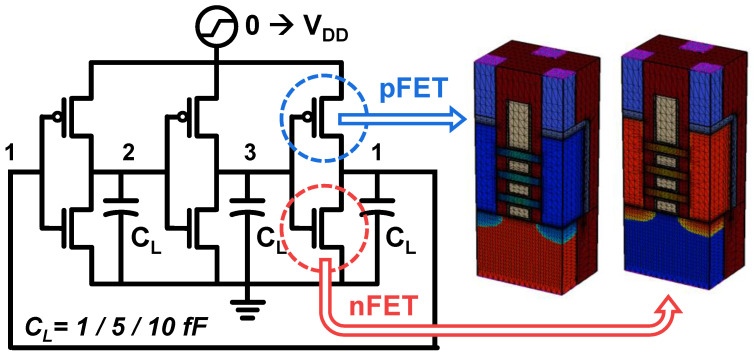
Schematic representation of the 3D mixed-mode simulation for a three-stage ring oscillator.

Although a steady-state operation of devices offers insights into their characteristics, it can lead to an overestimation of the device temperature. In contrast to steady-state operation, where the cooling of the device is not considered, a circuit operate under transient conditions cycles through heating and cooling periodically. Therefore, circuit-level performance evaluation is essential to compare the electro-thermal performance of the four schemes. In Section B, the NSFET-based three-stage ring oscillator (RO) is simulated using 3D mixed-mode simulation (Figure 10) [15]. In mixed-mode simulation, the process-simulated devices are first integrated directly into the netlist of the circuit, and then the equations for the devices and the circuit are solved numerically at the same time. Despite the high CPU time required for this, the 3D mixed-mode simulation delivers much more accurate results than the compact model-based SPICE circuit simulation. In particular, the SHE-related physical effects on the transistor, such as the increased carrier density and reduced *κ* at an elevated temperature, are still not accurately captured in compact SPICE models.

**Figure 11:**
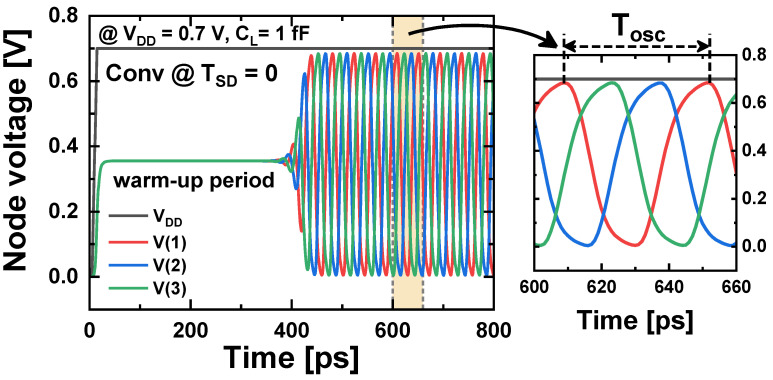
Transient voltage waveforms in a 3-stage RO consisting of Conv-NSFETs with *T_SD_* = 0.

**Figure 12:**
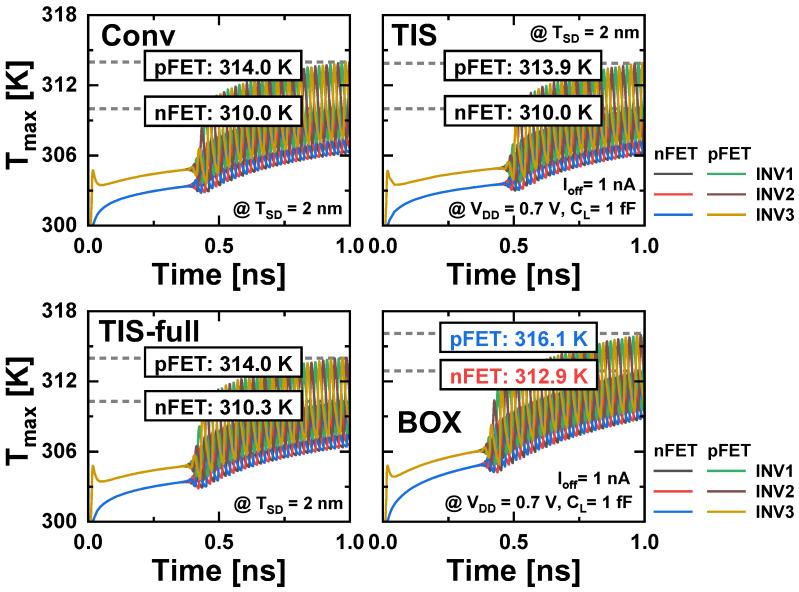
Transient *T_max_* response of the devices in each stage of the 3-stage RO.

Figure 11 illustrates the transient voltage waveforms in the three-stage RO. After *V_DD_* reaches its nominal value and a warm-up time of several hundred picoseconds has elapsed, the RO circuit begins to produce stable oscillation waveforms. The device temperature also oscillates with repeated heating and cooling in response to the transient voltage waveforms (Figure 12). In contrast to the *T_max_* under a steady-state condition where |*V_GS_*| and |*V_DS_*| are fixed at the *V_DD_* of 0.7 V and the cooling period is not considered, *T_max_* under the RO operation is much lower. This is because *V_GS_* and *V_DS_* oscillate rapidly within the range of 0 to *V_DD_*, and therefore, heat does not accumulate as much as in the steady state. Conv, TIS, and TIS-full schemes exhibit similar *T_max_* in 1 ns after RO operation (nFET: 314.0 K, pFET: 310.0 K). However, the BOX scheme still shows an approximately 2.6 and 2.1 K higher *T_max_* than the other schemes in n/pFET, respectively.

**Figure 13:**
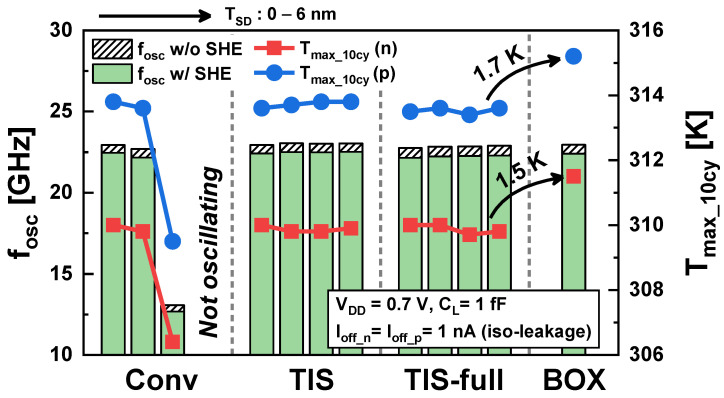
*f_osc_* and *T_max_10cy_* of the 3-stage RO in the Conv, TIS, TIS-full, and BOX schemes under the condition of iso-leakage.

For a more accurate comparison of electro-thermal performance between the four schemes, *f_osc_* and *T_max_* after 10 cycles (*T_max_10cy_*) after the end of the warm-up period are examined under an iso-leakage condition (*I_off_n_* = *I_off_p_* = 1 nA) (Figure 13). In the Conv scheme, *f_osc_* decreases drastically when *T_SD_* exceeds 2 nm, and *T_max_10cy_* also drops accordingly. Moreover, the RO with a *T_SD_* ≥ 6 nm does not oscillate due to the very low drive current. In contrast, in TIS and TIS-full schemes, the *f_osc_* and *T_max_10cy_* are almost retained regardless of the *T_SD_*. Comparing the TIS, TIS-full, and BOX schemes, they exhibit almost the same *f_osc_*. However, it is observed that *T_max_10cy_* is 1.5 K and 1.7 K higher in the BOX scheme for n/pFET, respectively.

**Figure 14:**
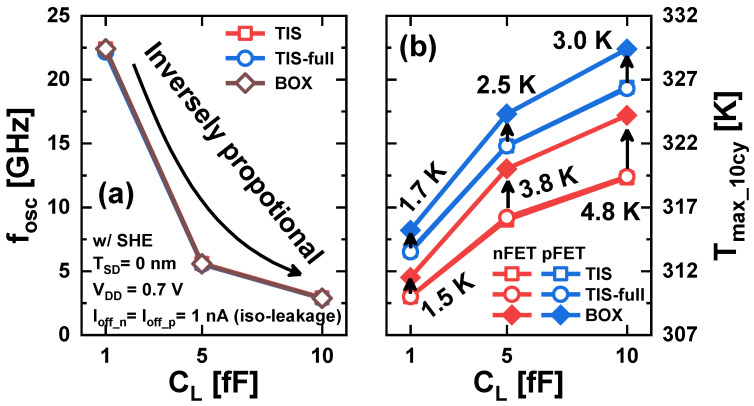
(**a**) *f_osc_* and (**b**) *T_max_10cy_* as a function of *C_L_* of the 3-stage RO in TIS, TIS-full, and BOX schemes (*f_osc_* is almost the same for all schemes, and they almost overlap).

On the other hand, in large-scale integration, many circuits are interconnected, so the load capacitance (*C_L_*) affecting the circuit operation is quite large. Therefore, the electro-thermal performance of RO under the larger *C_L_* should also be investigated (Figure 14). The *f_osc_* value is almost the same for each scheme and inversely proportional to *C_L_*. This is because the larger *C_L_* is, the larger the capacitance of the capacitor whose transistor must generate the potential change for the RO to oscillate. Alternatively, *T_max_10cy_* increases with increasing *C_L_* in all schemes. This is because a lower *f_osc_* increases the heating time, and the time required to cool the transistor to 300 K increases more than the increased heating time [26]. Consequently, more residual heat remains in the transistor when the *f_osc_* decreases. The TIS and TIS-full schemes exhibit almost the same *T_max_10cy_*, but the BOX schemes exhibit a higher *T_max_10cy_* than the others. Moreover, the *T_max_10cy_* differences become larger as the *C_L_* increases. The *T_max_10cy_* differences increase from 1.5 K (1.7 K) with *C_L_* = 1 fF to 4.8 K (3.0 K) with *C_L_* = 10 fF for n(p)FET. This implies that the electro-thermal performance superiority of the TIS scheme may become more evident in SoC applications where the scale and complexity of integrated circuits continue to increase. Therefore, an RO consisting of NSFETs with TIS and TIS-full schemes can achieve the same electrical performance at lower device temperatures compared to the BOX scheme. Moreover, this superiority of TIS and TIS-full schemes becomes even more evident in circuits with a larger *C_L_*.

## 4. Conclusions

In this study, an accurate and comprehensive evaluation of the electro-thermal performance of NSFETs with different *tr_pbt_*-controlling schemes ranging from single-device to three-stage RO circuits was performed. When evaluated for a single-device operation, the TIS scheme not only controlled the *tr_pbt_* effectively, but also had a lower *T_max_* than the BOX scheme because heat dissipation was not hindered in the TIS scheme. The *T_max_* of TIS-NSFETs was 59.6 K and 50.4 K lower than that of BOX-NSFETs in n/pFETs, respectively. However, the TIS scheme has the disadvantage that *C_gg_on_* increases with increasing *T_SD_*, resulting in a larger RC delay. This issue is particularly pronounced in pFETs, where the electro-thermal performance is worse than that of the BOX scheme when *T_SD_* exceeds 2 nm. Meanwhile, the TIS-full scheme, which increases the height of the TIS by removing SiGe_0.3_ residues below the TIS, enabled a reduction in *C_gg_on_* and exhibited the best electro-thermal performance among the various schemes under iso-leakage condition. Moreover, at the RO circuit level, the TIS and TIS-full schemes exhibited slightly lower *T_max_10cy_* while providing comparable *f_osc_* with the BOX scheme. However, the thermal superiority was more pronounced as the *C_L_* added to the RO becomes larger. When *C_L_* was 1 fF, the difference in *T_max_10cy_* of the TIS scheme was 1.5 and 1.7 K lower than that of the BOX scheme for n/pFETs, respectively. However, when *C_L_* increased to 10 fF, the difference grew to 4.8 and 3.0 K for n/pFETs, respectively. In conclusion, it is highly recommended to use the TIS or TIS-full scheme to simultaneously control *tr_pbt_* and improve electro-thermal performance in SoC products adapting sub-3 nm node NSFETs.

## Figures and Tables

**Table 1:** Geometrical and device parameters for sub-3 nm node NSFETs.

Parameters	Values	Parameters	Values
Contact poly pitch (CPP)	42 nm	Operating voltage (*V_DD_*)	0.7 V
Sheet pitch (SP)	60 nm	BOX thickness (*T_BOX_*)	10 nm
Gate length (*L_g_*)	12 nm	Contact resistivity	10^−9^ Ω·cm^2^
Spacing thickness (*T_sp_*)	10 nm	M0 resistivity	5 × 10^−5^ Ω·cm
Inner-spacer length (*L_is_*)	5 nm	Channel doping	1 × 10^15^ cm^−3^
NS width (*W_NS_*)	25 nm	S/D doping	5 × 10^20^ cm^−3^
NS thickness (*T_NS_*)	5 nm	PTS doping (Conv/TIS, BOX)	2 × 10^18^ cm^−3^
HfO_2_ thickness (*T_HK_*)	1.1 nm		1 × 10^15^ cm^−3^
Interfacial layer thickness (*T_IL_*)	0.6 nm	Over-etched S/D depth (*T_SD_*)	0–6 nm

**Table 2:** Thermal conductivities of the individual regions at 300 K.

Region	*κ* [W·m^−1^·K^−1^]	Region	*κ* [W·m^−1^·K^−1^]
Si substrate	170	HfO_2_	2.3
NS channel (n/p)	10.0/9.5	Low-*k*, BOX	0.7
PTS_Conv, TIS (n/p)	36.5/36.5	STI	1.4
PTS_BOX (n/p)	37.0/37.0	WFM	19.2
SiC_0.02_/SiGe_0.5_ S/D	30.0/13.5	Silicide	25
IL	1.4	M0	150

## Data Availability

Data are contained within the article.
